# Exploring the clinical significance of IL-38 correlation with PD-1, CTLA-4, and FOXP3 in colorectal cancer draining lymph nodes

**DOI:** 10.3389/fimmu.2024.1384548

**Published:** 2024-03-12

**Authors:** Liuhong Yuan, Zhenyu Tan, Junjie Huang, Feier Chen, Brett D. Hambly, Shisan Bao, Kun Tao

**Affiliations:** ^1^Department of Pathology, Tongji Hospital, Tongji University, Shanghai, China; ^2^Department of Pathology, Tongren Hospital, Shanghai Jiaotong University School of Medicine, Shanghai, China

**Keywords:** CRC, IL-38, PD-1, CTLA-4, Foxp3

## Abstract

**Introduction:**

Colorectal cancer (CRC) presents a substantial challenge characterized by unacceptably high mortality and morbidity, primarily attributed to delayed diagnosis and reliance on palliative care. The immune response of the host plays a pivotal role in carcinogenesis, with IL-38 emerging as a potential protective factor in CRC. However, the precise involvement of IL-38 among various leucocytes, its interactions with PD-1/PD-L1, and its impact on metastasis require further elucidation.

**Results:**

Our investigation revealed a significant correlation between IL-38 expression and metastasis, particularly concerning survival and interactions among diverse leucocytes within draining lymph nodes. In the mesentery lymph nodes, we observed an inverse correlation between IL-38 expression and stages of lymph node invasions (TNM), invasion depth, distance, and differentiation. This aligns with an overall survival advantage associated with higher IL-38 expression in CRC patients’ nodes compared to lower levels, as well as elevated IL-38 expression on CD4^+^ or CD8^+^ cells. Notably, a distinct subset of patients characterized by IL-38^high^/PD-1^low^ expression exhibited superior survival outcomes compared to other combinations.

**Discussion:**

Our findings demonstrate that IL-38 expression in colorectal regional nodes from CRC patients is inversely correlated with PD-1/PD-L1 but positively correlated with infiltrating CD4^+^ or CD8^+^ lymphocytes. The combined assessment of IL-38 and PD-1 expression in colorectal regional nodes emerges as a promising biomarker for predicting the prognosis of CRC.

## 1 Introduction

Colorectal cancer (CRC) remains a formidable challenge to human health, even with substantial technological advancements, especially in early malignancy screening over recent decades (1). The unacceptably high mortality and morbidity associated with CRC, particularly for individuals under the age of 50, have been on the rise globally. The multifaceted nature of tumorigenesis, shaped by genetic backgrounds (2), environmental factors (3), and the presence of infections or inflammation (4), underscores the inherent complexity of this disease.

Immunotherapy is intricately linked to the mismatch repair/microsatellite instability (MMR/MSI) status of tumours, which can be broadly divided into MMR/MSI competent tumours (representing the majority of CRCs) and MMR/MSI incompetent tumours (constituting around 4% of CRC cases) (5). Notably, MMR/MSI incompetent CRC represents an “immune-hot” subtype, characterized by a heightened tumour mutational burden, infiltration of T lymphocytes, and a robust anti-tumour immune response within the tumour microenvironment. Consequently, this CRC subtype exhibits a favourable response to immune checkpoint inhibitors (ICIs).

In contrast, the majority of CRCs are MMR/MSI competent, and their tumour growth is primarily propelled by increased WNT signalling (6). These tumours manifest an immune-exclusive microenvironment, likely due to a lower tumour mutational burden. As a result, MMR/MSI competent CRCs generally exhibit poor responses to ICIs. Nevertheless, ongoing clinical trials are exploring strategies to augment the inflammatory response within the tumour microenvironment of MMR/MSI competent CRCs. For instance, approaches such as radiotherapy are being investigated to induce inflammation and potentially enhance the responsiveness of this CRC subtype to ICIs (7).

Host immunity has been widely acknowledged as a critical determinant in the development of malignancies (8). Recent breakthroughs have prominently highlighted the pivotal role of the PD-1/PD-L1 axis in cancer progression (9). The introduction of anti-PD-1/PD-L1 therapies has heralded a paradigm shift in cancer treatment, presenting markedly improved outcomes for patients who would otherwise confront more severe conditions (10). Nevertheless, a notable proportion of adverse effects has been observed in cancer patients following anti-PD-1 therapy (11). For instance, our previous research demonstrated serious adverse responses in hepatocellular carcinoma patients in response to ICIs (11).

In our prior investigations, we provided evidence of the dichotomous effects of IL-36 (12) and IL-38 (13) on tumorigenesis of CRC, where IL-36 exhibited pro -tumour effects, while IL-38 displayed a contrasting impact. However, a critical gap in our understanding persists, particularly concerning the relationship among IL-38 expression, T or B cell infiltration, and PD-1/PD-L1 expression within the draining lymph nodes (LN) of patients with CRC. Our current study further investigates whether there is a correlation between IL-38 expression and metastasis, particularly in relation to the survival and the interaction among the different leucocytes within draining LN. These data is aligned with previous observations showing that the malignant subclones present within LNs exhibit a higher mutational burden and metastatic/proliferative potential than the primary tumour (14).

Such information serves as the impetus for the present study. Through a meticulous examination of IL-38 expression dynamics and its potential interplay with T and B cell infiltration, as well as the status of the PD-1/PD-L1 axis within the draining lymph nodes of CRC patients, we aim to unravel the intricacies of the immune landscape in this specific microenvironment. Such insights hold the promise of not only enhancing our comprehension of CRC immunobiology but also potentially identifying novel avenues for therapeutic intervention.

This research aims to more comprehensively define the immune modulators shaping the progression of CRC, with implications for the development of targeted therapies and personalized treatment strategies for individuals grappling with CRC.

## 2 Materials and methods

### 2.1 Demography of CRC patients and samples

The draining lymph nodes from colorectal regions in the wax blocks were obtained from CRC patients at the Department of Pathology, Tongren Hospital, Shanghai Jiaotong University School of Medicine. Demographic and clinicopathological information were extracted from the electronic medical database at Tongren Hospital. Follow-up data were retrieved from the *Centre for Disease Control and Prevention* in Changning District, Shanghai, China.

A total of 263 cases were included in the present study after excluding cases with incomplete clinical data and those where tissue specimens couldn’t be obtained for tissue array creation. Among these 263 cases, 231 had follow-up information available up to their date of death or their most recent contact, which was as of November 2023 (**Table 2**). Among these 231 CRC patients, 162 were still alive at the time of the analysis, while 69 had unfortunately died, with the longest survival period recorded at 52 months. This study has been approved by the human ethic committee, Tongren Hospital, Shanghai Jiaotong University.

All patients identified in the current study did not undergo preoperative neoadjuvant therapy and underwent surgery within two weeks of being diagnosed with CRC. Following surgery, patients who received chemotherapy in our hospital were prescribed capecitabine for a specific duration, with approximately two-thirds of patients also receiving oxaliplatin. Both capecitabine and oxaliplatin act by inhibiting the growth of tumour cells through the suppression of DNA synthesis in cancer cells (15, 16). There are no reported studies on capecitabine and oxaliplatin impact on the expression of IL-38. Additionally, targeted drugs, such as bevacizumab (17), used by a small subset of patients, have not been associated with any reported effects on IL-38 expression.

### 2.2 Immunohistochemistry

The tissue array was generated following the methods as described (18, 19). Subsequently, immunohistochemistry was conducted on sections obtained from these tissue arrays to assess the expression of IL-38, CD4, CD8, PD-1, CTLA-4, and FOXP3 on the draining lymph nodes containing metastatic CRC. All of the primary antibodies were purchased from Abcam, Cambridge, UK, details as follows: anti-human IL-38 (Ab180898, staining concentration 1:1000), anti-human CD4 (Ab133616, staining concentration 1:1000), anti-human CD8 (Ab237710, staining concentration 1:500), anti-human PD-1 (Ab237728, staining concentration 1:1000), anti-human CTLA-4 (Ab237712, staining concentration 1:400), and anti-human FOXP3 (Ab215206, staining concentration 1:200). A secondary HRP-conjugated antibody (Beijing Sequoia Jinqiao Biological Technology) was subsequently utilized. The specific target was visualized using a DAB detection kit and counterstained with hematoxylin.

To digitize the sections, we utilized NANO Zoomer series digital scanning devices 2.0 (Hamamatsu, Japan). Quantification of expression was conducted, using Halo digital imaging analysis software 2.0 (Indica Labs, USA). Notably, this software automatically excluded tissue gaps, including those resulting from prior tissue microarray coring, from the analysis. The images underwent annotation, and a staining intensity threshold was established, classifying them into negative and positive categories. Subsequently, the software employed an annotated training algorithm to automatically analyse and calculate staining intensity and the proportion of positive cells per unit area at each tumour and stromal site. The H score, combining staining intensity and the proportion of positive cells per unit area, served as a comprehensive representation of the results (Indica Labs, USA).

### 2.3 Immunofluorescent staining

To ascertain the co-localization of IL-38 with CD3, CD19, CD138, or CD68, we conducted immunofluorescence staining on the sections. The sections were stained with antibodies for anti-IL-38 (Ab180898, Abcam, Cambridge, UK) and anti-CD3 (MX036, Fuzhou Maixin Biological Technology, China), anti-CD19 (MX016, Fuzhou Maixin Biological Technology, China), anti-CD138 (MI15, Fuzhou Maixin Biological Technology, China), or CD68 (MX075, Fuzhou Maixin Biological Technology, China) at 4°C overnight. Subsequently, the sections were incubated with the corresponding fluorescent secondary antibodies: anti-IL-38 antibody (staining concentration 1:200) with its counterpart from Beijing Panovue Biological Technology, China, and CD3 (staining concentration: Ready-to-use concentration), CD19 (staining concentration: Ready-to-use concentration), CD138 (staining concentration: Ready-to-use concentration), or CD68 (staining concentration: Ready-to-use concentration) with their respective fluorescent secondary antibodies from Beijing Panovue Biological Technology, China. DAPI (Beijing Panovue Biological Technology, China) was applied to identify the nuclei. Co-localization was detected using a BX60 Olympus fluorescence microscope, and quantitative analysis was performed using Halo digital imaging analysis software 2.0 (Indica Labs, USA).

### 2.4 Statistical analysis

Statistical analysis was conducted following established procedures (20) utilizing Graphpad Prism 9.0.1 The comparison between two unpaired groups employed the Mann-Whitney U test, while comparisons among multiple groups utilized the Kruskal-Wallis H test. Optimal cut-off points for continuous variables were determined using X-tile software, based on the highest χ^2^ values defined by Kaplan-Meier survival analysis and the log-rank test (21). The patient’s overall survival was defined as the number of days between surgery and the date of the last follow-up or death. Survival curves were generated using the Kaplan-Meier method and compared using the log-rank test. Univariate and multivariate analyses of various factors influencing patient prognosis were conducted using Cox’s proportional hazards model. Statistical significance was considered at P < 0.05.

## 3 Results

### 3.1 Demographic information for the patients

Demographic data for the 263 primary CRC patients, subjected to comprehensive analysis, are detailed in **Table 1**. Within this cohort, 57 individuals exhibited tumours measuring < 3cm, while 206 patients presented tumours of ≥ 3cm. Employing the tumour-lymph node-metastasis (TNM) classification outlined by the American Joint Committee on Cancer (AJCC) (22), the distribution revealed 148 cases classified as N0 (no tumour metastasis in regional lymph nodes), 92 as N1, and 23 as N2. Additionally, the staging analysis disclosed 42 cases at stage I, 104 at stage II, 100 at stage III, and 17 at stage IV.

**Table 1:** Demography of patients with CRC.

Characteristics	Patients with CRC (n)
Sex	
Male	167
Female	96
Age (years)	
≤ 70	143
> 70	120
Position	
Left-sided	191
Right-sided	72
Size (diameter, cm)	
≤ 3	57
> 3	206
Differentiation	
Well	45
Moderate	188
Poor	30
Invasion depth	
T1	10
T2	47
T3	153
T4	53
Lymph node metastasis	
N0	148
N1	92
N2	23
Distant metastasis	
M0	246
M1	17
Stage (TNM)	
I	42
II	104
III	100
IV	17

**Table 2:** Association between IL-38 expression in colorectal reginal nodes and clinicopathological characteristics of patients.

Characteristics	IL-38 expression (H score)		*P*-value
	N (%)	median	
Sex			
Male	167 (63.5)	29.4	NS
Female	96 (36.5)	29.0	
Age (years)			
≤ 70	143 (54.4)	29.2	NS
> 70	120 (45.6)	29.9	
Position			
Left	191 (72.6)	27.3	NS
Right	72 (27.4)	31.9	
Size (diameter, cm)			
≤ 3	57 (21.7)	35.6	0.0386
> 3	206 (78.3)	27.2	
Differentiation			
Well	45 (17.1)	26.2	NS
Moderate	188 (71.5)	29.5	
Poor	30 (11.4)	32.6	
Invasion depth			
T1	10 (3.8)	36.0	NS
T2	47 (17.9)	33.6	
T3	153 (58.2)	26.8	
T4	53 (20.1)	27.3	
MLN metastasis			
N0	148 (56.3)	29.4	All: NS
N1	92 (35.0)	28.9	N0/N2:0.0415
N2	23 (8.7)	23.9	N0/N1: NS
			N1/N2: NS
Distant metastasis			
M0	246 (93.5)	29.4	NS
M1	17 (6.5)	23.7	
Stage (TNM)			
I	42 (16.0)	34.8	All: NS
II	104 (39.5)	26.7	I/IV:0.0413
III	100 (38.0)	28.9	I/II: NS
IV	17 (6.5)	23.7	I/III: NS
			II/IV: NS
			III/IV: NS

P values for Wilcoxon signed-rank test and Mann–Whitney U test.

#### 3.1.1 Association between IL-38 and clinicopathological characteristics in lymphoid tissue

**Figure 1:**
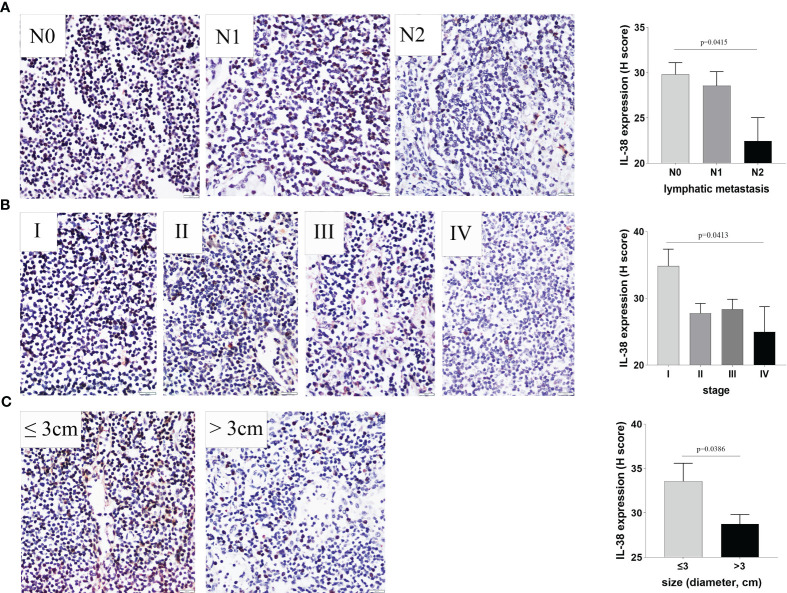
The Relationship between IL-38 expression in colorectal regional nodes and key clinicopathological factors. This figure illustrates the correlation between IL-38 expression in colorectal regional nodes and pertinent clinicopathological factors, including **(A)** the number of colorectal regional lymph nodes invaded by the tumour, **(B)** CRC stage, and **(C)** primary tumour size. Accompanying microphotographs depicting IL-38 expression are provided for visual reference. Original magnification ×400.

The expression of IL-38 was observed to be widespread within lymphoid tissues, primarily localized in the cytoplasm and nucleus of lymphocytes (**Figure 1**). Notably, as the number of regional lymph nodes invaded by the tumour increased, there was a discernible decrease in IL-38 expression in regional lymphoid tissue (**Figure 1A**; p=0.0415). Additionally, IL-38 exhibited a significant reduction in colorectal regional lymph nodes among patients with advanced CRC compared to those with early CRC (**Figure 1B**; p=0.0413). Lastly, IL-38 expression was markedly lower in the colorectal regional lymph nodes of patients with primary tumour sizes > 3 cm, in contrast to those in the regional lymph nodes of patients with primary tumour sizes of ≤ 3 cm (**Figure 1C**; p=0.0386).

#### 3.1.2 Co-localization of IL-38 in colorectal reginal nodes

**Figure 2:**
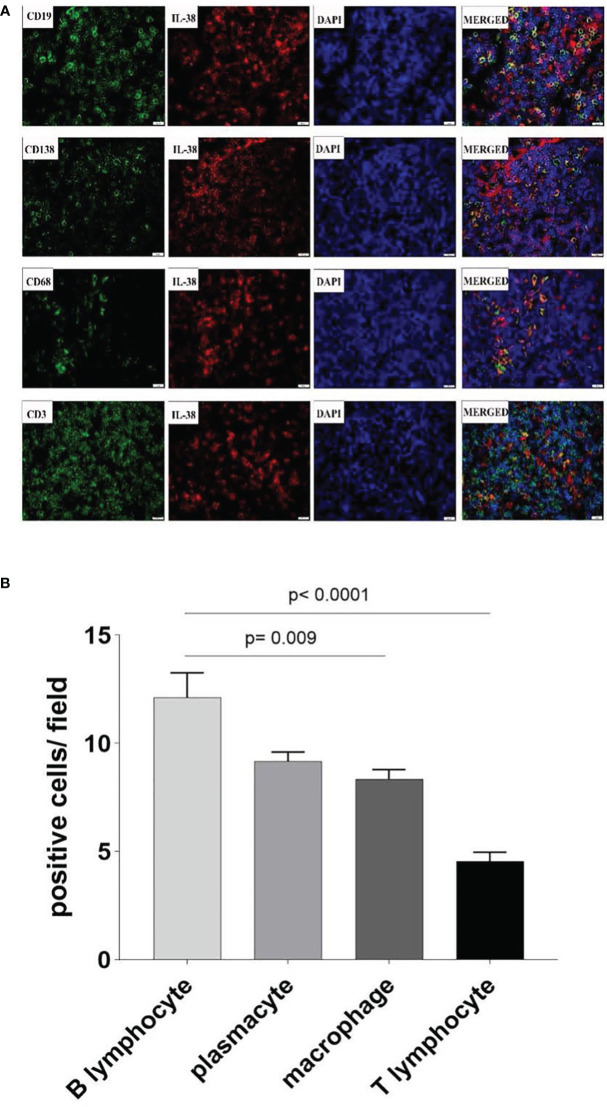
Co-localization of IL-38 in colorectal regional nodes from CRC patients was assessed using dual-coloured immunofluorescence. This method enabled the evaluation of CD3 (T lymphocyte), CD19 (B lymphocyte), CD138 (plasmacyte), or CD68 (monocyte/macrophage) expression (in green fluorescence) alongside IL-38 (in red fluorescence) (**Figure 2A**). Original magnification ×400. The double positive IL-18/CD19., IL-38/CD138, IL-38/CD68, IL-38/CD3 cells are quantified in **Figure 2B**).

To further identify the source of the cellular origins of IL-38 production within colorectal regional nodes of CRC patients, sections were double stained with fluorescence-labelled anti-IL-38 and antibodies against CD3, CD19, CD138, or CD68 (**Figure 2**). A significant portion of IL-38^+^ cells (depicted by red fluorescence) exhibited co-staining with CD19^+^ B lymphocytes (depicted by green fluorescence), whereas a comparatively smaller fraction displayed co-staining with CD138^+^ plasmacytes, CD68^+^ monocytes/macrophages, or CD3^+^ T lymphocytes, each represented by green fluorescence. The visual analysis indicated that the number of CD19^+^ B lymphocytes per visual field was the highest, followed by CD138^+^ plasmacytes and CD68^+^ monocytes/macrophages, with the lowest observed for CD3^+^ T lymphocytes. Consequently, B lymphocytes, plasmacytes, and monocytes/macrophages were identified as the primary cellular sources of IL-38 in the lymphatic tissue of CRC patients.

#### 3.1.3 Association between IL-38 and CD4, CD8, PD-1, CTLA-4 or FOXP3 in colorectal reginal nodes from CRC patients

**Figure 3:**
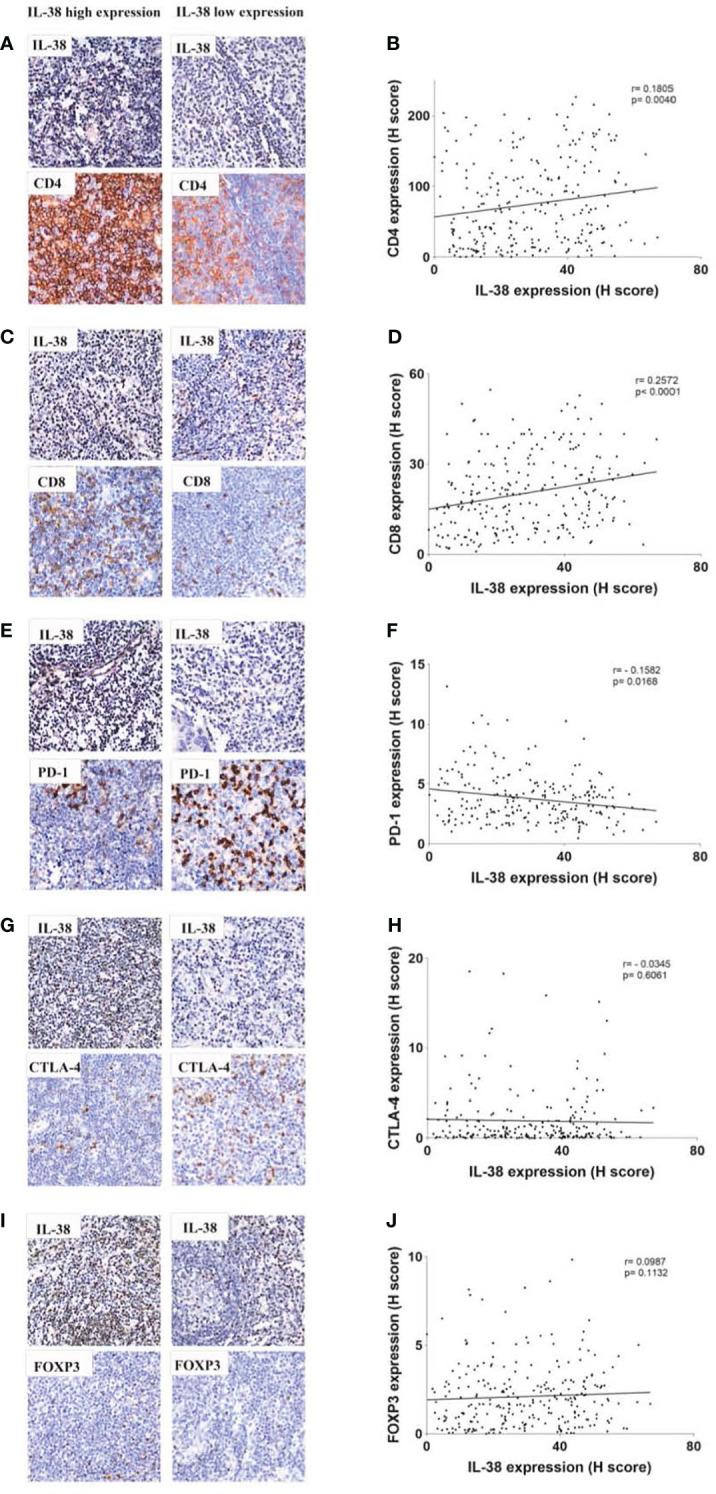
Correlation analysis between IL-38 and the expression of CD4, CD8, PD-1, CTLA-4, and FOXP3 in colorectal regional modes from patients with CRC. This figure presents a comprehensive correlation analysis between IL-38 and the expression of CD4, CD8, PD-1, CTLA-4, and FOXP3 in colorectal regional nodes from patients with CRC. Accompanying microphotographs offer visual representations of the correlations, specifically illustrating IL-38 co-staining with CD4 **(A)**, CD8 **(C)**, PD-1 **(E)**, CTLA-4 **(G)**, and FOXP3 **(I)**. Original magnification ×400. The correlation between CD4 and IL-38 **(B)**, IL-38 and CD8 **(D)**, IL-38 and PD-1 **(F)**, IL-38 and CTLA-4 **(H)** and IL-38 and FOXP3 **(J)** are presented.

In our examination of the relationship between IL-38 and CD4 or CD8 expression in colorectal regional nodes, positive correlations emerged between IL-38 and CD4 (r=0.1805, p=0.004) (**Figures 3A, B**) as well as between IL-38 and CD8 (r=0.2572, p<0.0001) (**Figures 3C, D**). Conversely, an inverse correlation was observed between IL-38 and PD-1 (r=-0.1582, p=0.0168) (**Figures 3E, F**). However, no significant correlation was discerned between IL-38 and CTLA-4 (r=-0.0345, p=0.6061) (**Figures 3G, H**) or between IL-38 and FOXP3 (r=0.0575, p=0.3554) (**Figure 3I, J**).

#### 3.1.4 Association IL-38, CD4, CD8, PD-1 and survival curves in CRC patients

We utilized the log-rank test to investigate the correlation between IL-38 expression in colorectal regional nodes and post-operative survival in CRC patients. Our results revealed a significant increase in overall survival rates among CRC patients exhibiting IL-38^high^ expression in colorectal regional nodes, as opposed to those with IL-38^low^ expression (**Figure 3A**; p=0.0436). The results also showed that patients with high CD4^high^ expression had a better prognosis than those with CD4^low^ expression (**Figure 3B**; p=0.0386), and conversely, patients with PD-1^low^ expression had a better prognosis (**Figure 3D**; p=0.0287).

Further sub-group analysis of CRC patients yielded insightful observations. Those with IL-38^low^ and PD-1^high^ expression in colorectal regional nodes displayed a markedly diminished survival rate compared to counterparts with IL-38^high^ and PD-1^low^ expression, or those with concurrent IL-38 and PD-1 high or low expression (**Figure 3G**; p=0.0093). In addition, the 5-year survival rate of patients with both IL-38 and CD4 high expression was higher than that of patients with double low expression (**Figure 3E**; p=0.0419).

**Figure 4:**
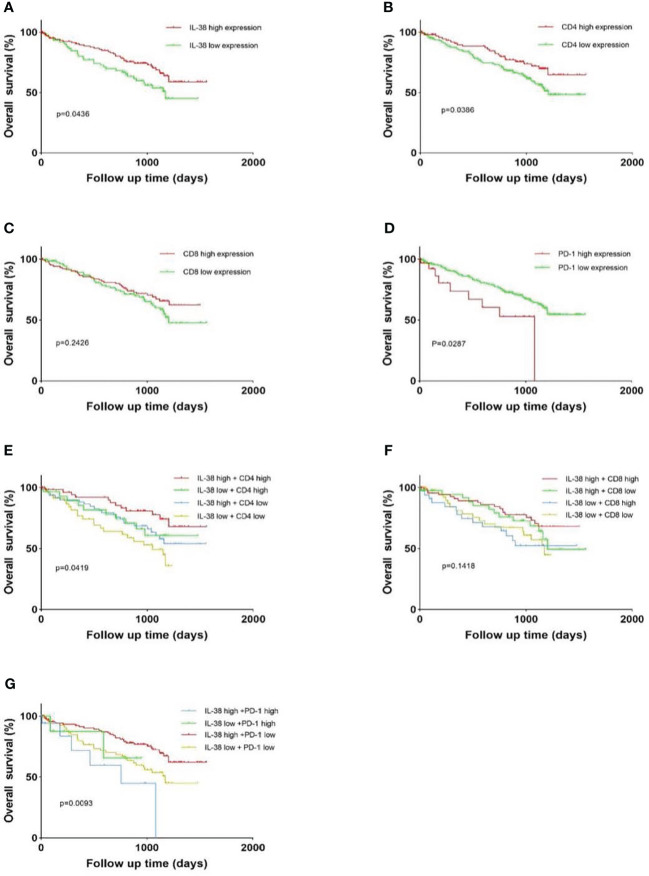
Kaplan-Meier survival curves depict patient outcomes based on high or low levels of expression for IL-38, CD4, CD8, and PD-1. Survival analysis for patients with IL-38, CD4, CD8 and PD-1 expression are illustrated in **(A–D)**. Survival analysis for patients with combinations of IL-38 and CD4 expression is shown in **(E)**, IL-38 and CD8 expression in **(F)**, and IL-38 and PD-1 expression in **(G)**. P values obtained from analysis were determined using the log-rank test.

However, CD8 expression was not significantly associated with patient prognosis (**Figure 3C**; p=0.2426), whether CD8 was analysed alone or in combination with IL-38, there was no significant relationship between their expression levels and patient prognosis (**Figure 3C**, p=0.2426; **Figure 3F**, p=0.1418) (**Figure 4**).

### 3.2 Univariate and multivariate analyses of the relationship between survival of CRC patients and IL-38, CD4, CD8, PD-1

Univariate analysis was conducted to assess the contribution of various factors (including IL-38, CD4, CD8, PD-1, sex, age, CRC location, tumour size, differentiation, depth of invasion, metastasis, and TNM staging) to the prediction of survival rates (**Table 3**). Both univariate and multivariate analyses were performed to determine CRC survival rates, as described (20). The results indicated that IL-38 expression (HR 1.626; 95% CI 1.009 - 2.618; p=0.046), CD4 expression (HR 1.636; 95% CI 1.021-2.622; p=0.041), PD-1 expression (HR 0.468; 95% CI 0.233-0.939; p=0.033), the combination of IL-38 and CD4 expression (HR 2.234; 95% CI 0.907-5.491; p=0.020), the combination of IL-38 and PD-1 expression (HR 0.500; 95% CI 0.309-0.807; p=0.005); age (HR 0.521; 95% CI 0.337-0.804; p=0.003), lymph node metastasis (HR 0.738; 95% CI 0.277 - 1.772; p=0.021), distant metastasis (HR 0.261; 95% CI 0.147 - 0.466; p<0.001), and TNM staging (HR 0.236; 95% CI 0.063 - 0.619; p<0.001) were all significant predictors of survival among CRC patients in the univariate analysis.

**Table 3:** Univariate and multivariate analysis of IL-38, CD4, CD8, PD-1 and clinicopathological features affecting survival of patients with CRC.

Characteristics	Univariate analysis		Multivariate analysis	
	*HR* (95% *CI*)	*P* value	*HR* (95% *CI*)	*P* value
Sex				
Male/female	1.138 (0.730-1.773)	NS		
Age (years)				
<70/≥70	0.521 (0.337-0.804)	0.003	0.611 (0.367 - 1.018)	NS
Position				
Right-sided/left-sided	0.867 (0.554-1.356)	NS		
Size (diameter, cm)				
≤3/>3	0.586 (0.337-1.022)	NS		
Differentiation				
Well /moderate/poor	0.530 (0.262-1.135)	NS		
Invasion depth				
T1/T2/T3/T4	0.485 (0.019 - 1.435)	NS		
Lymph node metastasis				
No/yes	0.738 (0.277 - 1.772)	0.021	0.997 (0.180 - 7.344)	NS
Distant metastasis				
No/yes	0.261 (0.147 - 0.466)	< 0.001	1.175 (0.090 - 0.340)	< 0.001
TNM				
I/II/III/IV	0.236 (0.063 - 0.619)	< 0.001	0.161 (0.017 – 0.726)	NS
IL-38				
High/low	1.626 (1.009 - 2.618)	0.046	0.514 (0.090 – 2.920)	NS
CD4				
High/low	1.636 (1.021-2.622)	0.041	0.353 (0.152 – 0.804)	NS
CD8				
High/low	1.294 (0.825-2.031)	NS		
PD-1				
High/low	0.468 (0.233-0.939)	0.033	0.469 (0.211 – 1.044)	NS
IL-38 and CD4				
DH/ SH/ DL	2.234 (0.907-5.491)	0.020	2.003 (0.771 – 6.815)	NS
IL-38 and CD8				
SH1/ SH2/ DH/ DL	1.780 (0.684-4.234)	NS		
IL-38 and PD-1				
SH1/SH2 or DH or DL	0.500 (0.309-0.807)	0.005	0.587 (0.351 – 0.984)	0.043

DH, double high expression; DL, double low expression; SH, single high expression; SH1, IL-38 high and CD8 low/ PD-1 low expression; SH2, IL-38 low and CD8 high/ PD-1 high expression.

Notably, the multivariate analysis revealed that the combination of IL-38 and PD-1 expression (HR 0.587; 95% CI 0.351 – 0.984; p=0.043) and distant metastasis (HR 0.175; 95% CI 0.090 - 0.340; p<0.001) emerged as independent and reliable biomarkers for predicting survival rates among CRC patients (**Table 3**). In contrast, other factors such as age, TNM staging and lymph node metastasis did not exhibit significant predictive value for survival rates among these CRC patients.

## 4 Discussion

In the present study, we investigated the correlation between IL-38 expression, T cell subsets, and the source of IL-38 within colorectal regional nodes from CRC patients. We observed an inverse correlation between IL-38 expression and the number of colorectal regional nodes invaded by tumour in patients with CRC. Furthermore, there was an inverse correlation between IL-38 expression in colorectal regional nodes and CRC TNM stages. Additionally, IL-38 exhibited an inverse correlation with PD-1 expression while demonstrating a positive correlation with the numbers of CD4^+^ and CD8^+^ T cells in colorectal regional nodes. Significantly, our multivariate analysis identified high levels of IL-38^high^ and PD-1^low^ as two independent predictors, which can serve as valuable biomarkers for predicting prognosis in CRC patients.

IL-38, recognized as an anti-inflammatory cytokine, plays a crucial role in preserving local host immunity (22) and contributing to the homeostasis of mucosal function (23). In the context of dysregulated intestinal mucosal immunity, particularly in chronic intestinal inflammation prone to the development of CRC (24), our findings are in line with a prior study indicating a correlation between IL-38 expression in CRC tissue and patient survival. This suggests that IL-38 may afford protection during the progression of CRC patients (13). Additionally, our observations align with other studies indicating that exogenous IL-38 inhibits the proliferation and metastasis of CRC cells *in vitro* (25).

Our present study serves as an extension of our prior research, with a specific focus on IL-38 within the context of gut mucosal immunity. Concentrating on the draining lymph nodes, our investigation reveals an inverse correlation between IL-38 expression and the invasion or metastasis of CRC in colorectal regional nodes from CRC patients. This finding lends support to the notion that IL-38 may confer benefits to CRC patients by suppressing invasion and metastasis.

The majority of CRC patients with poor prognoses often face challenges related to local and/or distant metastasis (23), Notably, we observed a significantly lower expression of IL-38 in colorectal regional nodes from CRC patients with advanced stages of the disease compared to those at an early stage. This observation aligns with the extent of colorectal regional nodes invaded by tumours in our current study. Such consistency in our findings further reinforces the protective role of IL-38 in the development of CRC in real-world scenarios, emphasizing its potential as a promising therapeutic target in the management of CRC patients.

The origin of intestinal mucosal IL-38 has been identified in B lymphocytes in ulcerative colitis (26). Our investigation demonstrates that IL-38 is produced, in sequential order of level of production, by B lymphocytes, plasmacytes, macrophages, and T lymphocytes in colorectal regional nodes from CRC patients. The observed inverse correlation between intestinal IL-38 levels in lymph nodes and advanced CRC, as well as TNM stages, provides additional evidence supporting the protective role of IL-38 in the tumorigenesis of CRC.

This discovery prompts speculation that the host may be actively endeavouring to suppress the development of CRC within the metastatic tumour within regional nodes by secreting IL-38. However, it appears that the target cells may not respond optimally to the elevated IL-38 levels among these CRC patients. It remains to be elucidated whether a compromised IL-38 signalling pathway exists in these susceptible individuals (27). Notably, IL-38 expression exhibited a positive correlation with the expression of CD4^+^ or CD8^+^ T lymphocytes, indicating a potential influence of IL-38 and CD4 or CD8 in shaping the progression of CRC by modulating the tumour microenvironment during CRC pathogenesis.

This concept gains support from existing research, where IL-38 expression was significantly associated with CD8^+^ tumour-infiltrating lymphocytes in lung cancer (28), and IL-38 was shown to potentially impact the further differentiation of CD4^+^ T cells (29). We hypothesize that IL-38 might inhibit CRC metastasis by influencing the infiltration of CD4^+^ and CD8^+^ T lymphocytes, a hypothesis that warrants verification in subsequent experiments. Additionally, the reason for the observed decrease in IL-38 expression in colorectal regional nodes from CRC patients, particularly among those with multiple regional nodes metastasis, remains unclear. While IL-38 appears to play a protective role in CRC development, the precise pathogenesis involved in CRC is yet to be thoroughly explored.

The immune checkpoint mechanism is a pivotal factor enabling tumour cells to evade host immune system attacks (30). Our findings reveal that IL-38 expression is inversely correlated with PD-1, but not with CTLA-4 or FOXP3, in regional nodes from CRC patients. This suggests that IL-38 could be a potential target for precision medicine in CRC treatment, complementing anti-PD-1/PD-L1 antibody therapy. This proposition is reinforced by the positive outcomes observed in anti-PD-1/PD-L1 treatment reported by others (31).

Our data are supported by previous findings, showing that there are different components and/or pathways during the development of cancers, including CRC. It has been reported that the roles of CTLA-4 and PD-1 in inhibiting immune responses, including antitumor responses, are largely distinct. CTLA-4 is thought to regulate T-cell proliferation early in an immune response, primarily in lymph nodes, whereas PD-1 suppresses T cells later in an immune response, primarily in peripheral tissues (32). Thus, clinical interventions to manipulate these 2 checkpoints may vary based on their mechanistic differences.

Our study reveals an inverse correlation between IL-38 expression levels and PD-1 in CRC draining lymph nodes. Immunofluorescence double staining results indicate that IL-38 is primarily expressed in B lymphocytes and plasma cells. Numerous studies have validated the effective inhibition of cancer metastasis through PD-1 blockade. We propose that IL-38 might transform PD-1 positive CRC cells into PD-1 negative CRC cells, thereby inhibiting CRC development. This intriguing possibility awaits verification in future research endeavours (33). Furthermore, in regional lymph nodes, we speculate that IL-38 derived from B lymphocytes and plasma cells might suppress the expression of PD-1 on the cell membrane of T lymphocytes, thereby inhibiting cancer cell metastasis in the lymph nodes. Our data also demonstrate a correlation between the expression of IL-38 and CD8, suggesting a potential regulatory role for them during the development of CRC. This observation is partially aligned with the research conducted by Kinoshita et al. (28). Thus, we hypothesize that IL-38 may inhibit lymph node metastasis in CRC by increasing CD8^+^ T lymphocytes.

Nevertheless, no correlation was observed between IL-38 and CTLA-4 in regional nodes from CRC patients. We posit that this lack of correlation may stem from the distinct roles played by PD-1 and CTLA-4 during the development of CRC, especially in the subset of CRC patients with regional nodes metastasis. It has been reported that FOXP3^+^ Treg cells contribute to the progression of malignancy by regulating host immunity within the local microenvironment in these susceptible cohorts (34).

Furthermore, our investigation revealed a higher survival rate among CRC patients with IL-38^hi^ and PD-1^low^ expression in colorectal regional nodes compared to those with IL-38^low^ and PD-1^hi^ expression. The blockade of PD-1/PD-L1 has emerged as a revolutionary approach in the management of malignancies (35), including promising outcomes in CRC patients (36). Notably, our previous report established a correlation between colonic IL-38 expression and 5-year survival (13). Hence, it is reasonable to infer that our current finding, indicating a better prognosis for CRC patients with IL-38^hi^ and PD-1^low^ expression compared to those with IL-38^low^ and PD-1^hi^ expression, further underscores the crucial protective role of IL-38 in CRC development. This may be associated with its potential to regulate the expression of PD-1.

Classically, PD-1 is primarily expressed in lymphocytes, with PD-L1 being expressed on cancer cells. However, some studies indicate that PD-1 can also be expressed in certain cancer cells and promote tumour growth independent of adaptive immunity (37–39). Searching *The Cancer Genome Atlas* (TCGA) database reveals widespread transcription of the *PDCD1* gene, which encodes PD-1, in 17 cancers including CRC (33). Additionally, CRC cell lines have shown varying degrees of PD-1 expression (33). Our studies indicate that colonic IL-38 serves as a protective factor, and its expression level exhibits an inverse correlation with PD-1. This suggests a potential mutual regulation between IL-38 and PD-1 during the development of CRC. Our speculation provides a valuable reference for further research in this direction.”

An intriguing observation is that IL-38 upregulates the number of circulating CD4^+^/CD25^+^/FOXP3^+^ Treg cells in sepsis patients, possibly reflecting the host’s effort to mitigate the cytokine storm in sepsis cases (40). While the literature lacks information on the role of FOXP3^+^ Treg cells in the context of CRC development, necessitating further investigation, our study reveals no significant correlation between IL-38 and FOXP3^+^ Treg cells in the mesenteric lymph nodes from CRC patients. Such observation suggests that FOXP3^+^ Treg cells may not play a significant role in the metastasis observed in the regional nodes from CRC patients.

Furthermore, we established a correlation between IL-38 expression in colorectal regional nodes and the prognosis of CRC patients, aligning with our earlier discovery in CRC tissues (13). This consistency implies the protective role of IL-38 in the progression of CRC. Notably, we observed variations in statistical power between regional nodes and CRC tissues, with a more pronounced and significant correlation identified in colorectal regional nodes. Our data suggest that colorectal regional nodes may exhibit greater sensitivity compared to CRC tissues, potentially linked to the heightened accumulation of lymphocytes in these nodes.

Our current study was an extension of our previous report, showing that a similar pattern of the expression of IL-38 in lymphoid tissue and the 5-year survival rate of patients (13). However, the advantage of current study lies in the expansion of sample size and an increased follow-up rate (88%). Additionally, determination of PD-1 enhances the sensitivity of IL-38 in predicting patient prognosis, indicating a mutual improvement in sensitivity. An inverse correlation is observed between IL-38 and PD-1, providing some guidance for the application of immune checkpoint inhibitors in patients.

Our previous study has demonstrated that IL-38 is inhibited in CRC tissues, correlating with survival (13). This suggests that colonic IL-38 plays a protective role during the development of CRC. In contrast, IL-38 promotes lung cancer through the infiltration of CD8^+^ T cells (28). The divergent roles of IL-38 in CRC and lung cancers may be attributed to the significant differences in microbial flora loads (>1000 fold) between these two organs (41). These variations could trigger distinct host immune responses, leading to entirely different regulatory functions in the colon and lungs, despite both organs being safeguarded by mucosal-associated lymphoid organs.

We identified an inverse correlation between IL-38 and PD-1 expression in the lymph nodes of colorectal cancer patients. This discovery suggests that IL-38 levels may serve as a potential predictive factor for outcomes in CRC patients undergoing PD-1/PD-L1 therapy.

There are some limitations from the current study, for example, we should utilize Western blot and/or qRT-PCR to illustrate the possible signalling pathway, in addition to our current immunohistochemistry, which is currently being investigated. We also should extend our study into multiple centres and if possible, within different regions and/or countries with different racial backgrounds.

In conclusion, our data demonstrated that the expression of IL-38 in colorectal reginal nodes from the CRC patients was inversely correlated with PD-1/PD-L1, but positively correlated with infiltrating CD4^+^ or CD8^+^ lymphocytes. The combination of IL-38 and PD-1 expression in colorectal reginal nodes from CRC patients seems to be a good biomarker in predicting prognosis of CRC.

## Data availability statement

The original contributions presented in the study are included in the article/supplementary material. Further inquiries can be directed to the corresponding authors.

## Ethics statement

The studies involving humans were approved by Tongren Hospital Human Ethic Committee. The studies were conducted in accordance with the local legislation and institutional requirements. The participants provided their written informed consent to participate in this study.

## Author contributions

LY: Writing – original draft, Methodology, Formal Analysis, Data curation. ZT: Writing – review & editing, Methodology. JH: Writing – review & editing, Project administration. FC: Writing – review & editing, Methodology. BH: Writing – review & editing, Validation. SB: Writing – review & editing, Conceptualization. KT: Writing – review & editing, Supervision, Resources, Funding acquisition.

## References

[1] Patel SG Karlitz JJ Yen T Lieu CH Boland CR. The rising tide of early-onset colorectal cancer: a comprehensive review of epidemiology, clinical features, biology, risk factors, prevention, and early detection. Lancet Gastroenterol Hepatol. (2022) 7:262–74. doi: 10.1016/S2468-1253(21)00426-X 35090605

[2] Ma H Brosens LAA Offerhaus GJA Giardiello FM de Leng WWJ Montgomery EA. Pathology and genetics of hereditary colorectal cancer. Pathology. (2018) 50:49–59. doi: 10.1016/j.pathol.2017.09.004 29169633

[3] Sedlak JC Yilmaz Ö H Roper J. Metabolism and colorectal cancer. Annu Rev Pathol. (2023) 18:467–92. doi: 10.1146/annurev-pathmechdis-031521-041113 PMC9877174 36323004

[4] Song M Chan AT Sun J. Influence of the gut microbiome, diet, and environment on risk of colorectal cancer. Gastroenterology. (2020) 158:322–40. doi: 10.1053/j.gastro.2019.06.048 PMC6957737 31586566

[5] Hirano H Takashima A Hamaguchi T Shida D Kanemitsu Y. Current status and perspectives of immune checkpoint inhibitors for colorectal cancer. Jpn J Clin Oncol. (2021) 51:10–9. doi: 10.1093/jjco/hyaa200 33205813

[6] Schatoff EM Leach BI Dow LE. Wnt signaling and colorectal cancer. Curr Colorectal Cancer Rep. (2017) 13:101–10. doi: 10.1007/s11888-017-0354-9 PMC5391049 28413363

[7] Parikh AR Szabolcs A Allen JN Clark JW Wo JY Raabe MZ. Radiation therapy enhances immunotherapy response in microsatellite stable colorectal and pancreatic adenocarcinoma in a phase II trial. Nat Cancer. (2021) 2:1124–35. doi: 10.1038/s43018-021-00269-7 PMC8809884 35122060

[8] Hendry SA Farnsworth RH Solomon B Achen MG Stacker SA Fox SB. The role of the tumor vasculature in the host immune response: implications for therapeutic strategies targeting the tumor microenvironment. Front Immunol. (2016) 7:621. doi: 10.3389/fimmu.2016.00621 28066431 PMC5168440

[9] Han Y Liu D Li L. PD-1/PD-L1 pathway: current researches in cancer. Am J Cancer Res. (2020) 10:727–42. PMC7136921 32266087

[10] Sanmamed MF Chen L. A paradigm shift in cancer immunotherapy: from enhancement to normalization. Cell. (2018) 175:313–26. doi: 10.1016/j.cell.2018.09.035 PMC6538253 30290139

[11] Lou S Cao Z Chi W Wang X Feng M Lin L. The safety concerns regarding immune checkpoint inhibitors in liver cancer patients rising mainly from CHB. Front Pharmacol. (2023) 14:1164309. doi: 10.3389/fphar.2023.1164309 37168999 PMC10165088

[12] Chen F Qu M Zhang F Tan Z Xia Q Hambly BD. IL-36 s in the colorectal cancer: is interleukin 36 good or bad for the development of colorectal cancer? BMC Cancer. (2020) 20:92. doi: 10.1186/s12885-020-6587-z 32013927 PMC6998229

[13] Chen F Zhang F Tan Z Hambly BD Bao S Tao K. Interleukin-38 in colorectal cancer: a potential role in precision medicine. Cancer Immunol Immunother. (2020) 69:69–79. doi: 10.1007/s00262-019-02440-7 31786620 PMC11027872

[14] Puccini A Seeber A Xiu J Goldberg RM Soldato D Grothey A. Molecular differences between lymph nodes and distant metastases compared with primaries in colorectal cancer patients. NPJ Precis Oncol. (2021) 5:95. doi: 10.1038/s41698-021-00230-y 34707195 PMC8551277

[15] Jardim DL Rodrigues CA Novis YAS Rocha VG Hoff PM. Oxaliplatin-related thrombocytopenia. Ann Oncol. (2012) 23:1937–42. doi: 10.1093/annonc/mds074 22534771

[16] García-Alfonso P Muñoz Martín AJ Ortega Morán L Soto Alsar J Torres Pérez-Solero G Blanco Codesido M. Oral drugs in the treatment of metastatic colorectal cancer. Ther Adv Med Oncol. (2021) 13:17588359211009001. doi: 10.1177/17588359211009001 33995592 PMC8111515

[17] Rosen LS Jacobs IA Burkes RL. Bevacizumab in colorectal cancer: current role in treatment and the potential of biosimilars. Target Oncol. (2017) 12:599–610. doi: 10.1007/s11523-017-0518-1 28801849 PMC5610666

[18] Chen L Zhu C Li F Wang Y Bao R Cao Z. Correlation between hepatic human males absent on the first (hMOF) and viral persistence in chronic hepatitis B patients. Cell Biosci. (2018) 8:14. doi: 10.1186/s13578-018-0215-5 29484170 PMC5819663

[19] Zhou T Sun Y Li M Ding Y Yin R Li Z. Enhancer of zeste homolog 2-catalysed H3K27 trimethylation plays a key role in acute-on-chronic liver failure via TNF-mediated pathway. Cell Death Dis. (2018) 9:590. doi: 10.1038/s41419-018-0670-2 29789597 PMC5964223

[20] Cao Z Li Z Wang H Liu Y Xu Y Mo R. Algorithm of Golgi protein 73 and liver stiffness accurately diagnoses significant fibrosis in chronic HBV infection. Liver Int. (2017) 37:1612–21. doi: 10.1111/liv.13536 28772348

[21] Jiang Y Zhu Y Xiang Z Sasmita BR Wang Y Ming G. The prognostic value of admission D-dimer level in patients with cardiogenic shock after acute myocardial infarction. Front Cardiovasc Med. (2022) 9:1083881. doi: 10.3389/fcvm.2022.1083881 36698952 PMC9868698

[22] van de Veerdonk FL de Graaf DM Joosten LA Dinarello CA. Biology of IL-38 and its role in disease. Immunol Rev. (2018) 281:191–6. doi: 10.1111/imr.12612 29247986

[23] Wang J Li S Liu Y Zhang C Li H Lai B. Metastatic patterns and survival outcomes in patients with stage IV colon cancer: A population-based analysis. Cancer Med. (2020) 9:361–73. doi: 10.1002/cam4.2673 PMC6943094 31693304

[24] Ullman TA Itzkowitz SH. Intestinal inflammation and cancer. Gastroenterology. (2011) 140:1807–16. doi: 10.1053/j.gastro.2011.01.057 21530747

[25] Huang L Zhang H Zhao D Hu H Lu Z. Interleukin-38 suppresses cell migration and proliferation and promotes apoptosis of colorectal cancer cell through negatively regulating extracellular signal-regulated kinases signaling. J Interferon Cytokine Res. (2021) 41:375–84. doi: 10.1089/jir.2021.0047 34612721

[26] Xie C Yan W Quan R Chen C Tu L Hou X. Interleukin-38 is elevated in inflammatory bowel diseases and suppresses intestinal inflammation. Cytokine. (2020) 127:154963. doi: 10.1016/j.cyto.2019.154963 31927461

[27] Diaz-Barreiro A Huard A Palmer G. Multifaceted roles of IL-38 in inflammation and cancer. Cytokine. (2022) 151:155808. doi: 10.1016/j.cyto.2022.155808 35066449

[28] Kinoshita F Tagawa T Akamine T Takada K Yamada Y Oku Y. Interleukin-38 promotes tumor growth through regulation of CD8(+) tumor-infiltrating lymphocytes in lung cancer tumor microenvironment. Cancer Immunol Immunother. (2021) 70:123–35. doi: 10.1007/s00262-020-02659-9 PMC10991933 32653939

[29] Lai M Peng H Wu X Chen X Wang B Su X. IL-38 in modulating hyperlipidemia and its related cardiovascular diseases. Int Immunopharmacol. (2022) 108:108876. doi: 10.1016/j.intimp.2022.108876 35623295

[30] Pang K Shi ZD Wei LY Dong Y Ma YY Wang W. Research progress of therapeutic effects and drug resistance of immunotherapy based on PD-1/PD-L1 blockade. Drug Resist Update. (2023) 66:100907. doi: 10.1016/j.drup.2022.100907 36527888

[31] Zhang X Yang Z An Y Liu Y Wei Q Xu F. Clinical benefits of PD-1/PD-L1 inhibitors in patients with metastatic colorectal cancer: a systematic review and meta-analysis. World J Surg Oncol. (2022) 20:93. doi: 10.1186/s12957-022-02549-7 35331250 PMC8944161

[32] Buchbinder EI Desai A. CTLA-4 and PD-1 pathways: similarities, differences, and implications of their inhibition. Am J Clin Oncol. (2016) 39:98–106. doi: 10.1097/COC.0000000000000239 26558876 PMC4892769

[33] Ieranò C Righelli D D'Alterio C Napolitano M Portella L Rea G. In PD-1+ human colon cancer cells NIVOLUMAB promotes survival and could protect tumor cells from conventional therapies. J Immunother Cancer. (2022) 10:1–14. doi: 10.1136/jitc-2021-004032 PMC8900051 35246475

[34] Whiteside TL Schuler P Schilling B. Induced and natural regulatory T cells in human cancer. Expert Opin Biol Ther. (2012) 12:1383–97. doi: 10.1517/14712598.2012.707184 PMC3730844 22849383

[35] Yu L Sun M Zhang Q Zhou Q Wang Y. Harnessing the immune system by targeting immune checkpoints: Providing new hope for Oncotherapy. Front Immunol. (2022) 13:982026. doi: 10.3389/fimmu.2022.982026 36159789 PMC9498063

[36] Yang Z Wu G Zhang X Gao J Meng C Liu Y. Current progress and future perspectives of neoadjuvant anti-PD-1/PD-L1 therapy for colorectal cancer. Front Immunol. (2022) 13:1001444. doi: 10.3389/fimmu.2022.1001444 36159842 PMC9501688

[37] Du S McCall N Park K Guan Q Fontina P Ertel A. Blockade of Tumor-Expressed PD-1 promotes lung cancer growth. Oncoimmunology. (2018) 7:e1408747. doi: 10.1080/2162402X.2017.1408747 29632720 PMC5889288

[38] Li H Li X Liu S Guo L Zhang B Zhang J. Programmed cell death-1 (PD-1) checkpoint blockade in combination with a mammalian target of rapamycin inhibitor restrains hepatocellular carcinoma growth induced by hepatoma cell-intrinsic PD-1. Hepatology. (2017) 66:1920–33. doi: 10.1002/hep.29360 28732118

[39] Kleffel S Posch C Barthel SR Mueller H Schlapbach C Guenova E. Melanoma cell-intrinsic PD-1 receptor functions promote tumor growth. Cell. (2015) 162:1242–56. doi: 10.1016/j.cell.2015.08.052 PMC4700833 26359984

[40] Ge Y Huang M Wu Y Dong N Yao Y. Interleukin-38 protects against sepsis by augmenting immunosuppressive activity of CD4(+) CD25(+) regulatory T cells. J Cell Mol Med. (2020) 24:2027–39. doi: 10.1111/jcmm.14902 PMC6991686 31880383

[41] Kumar V Abbas AK Aster JC. Robbins and cotran pathologic basis of disease ninth edition, Amsterdam, Netherlands. Vol. 1391. (2015).

